# TMEM106A transcriptionally regulated by promoter methylation is involved in invasion and metastasis of hepatocellular carcinoma: TMEM106A is involved in invasion and metastasis of HCC

**DOI:** 10.3724/abbs.2022069

**Published:** 2022-06-09

**Authors:** Shiming Shi, Biao Wang, Jinglei Wan, Lina Song, Guiqi Zhu, Junxian Du, Luxi Ye, Qianqian Zhao, Jialiang Cai, Qing Chen, Kun Xiao, Jian He, Lei Yu, Zhi Dai

**Affiliations:** 1 Liver Cancer Institute Zhongshan Hospital Fudan University & State Key Laboratory of Genetic Engineering Fudan University Shanghai 200032 China; 2 Department of Radiation Oncology Zhongshan Hospital Fudan University Shanghai 200032 China; 3 Department of Pediatric Surgery the First Affiliated Hospital of Sun Yat-sen University Guangzhou 510080 China; 4 Department of General Surgery Zhongshan Hospital (South) Fudan University Shanghai Public Health Clinical Center Fudan University Shanghai 200083 China; 5 Department of Gastrointestinal Surgery Shandong Provincial Hospital Affiliated to Shandong First Medical University Jinan 250021 China; 6 Department of Liver Surgery and Transplantation Liver Cancer Institute Zhongshan Hospital Fudan University Shanghai 200032 China

**Keywords:** hepatocellular carcinoma, transmembrane protein 106A, methylation, epithelial mesenchymal transition, metastasis

## Abstract

Expression of transmembrane protein 106A (TMEM106A) has been reported to be dysregulated in several types of cancers. However, the role of TMEM106A in hepatocellular carcinoma (HCC) is still unknown. In the present study, we demonstrate that TMEM106A is markedly downregulated in HCC compared with normal liver tissue. In particular, tumor-specific DNA methylation of TMEM106A is frequently observed in tumor tissues from HCC patients. Immunohistochemistry and pyrosequencing reveal a significant relationship between TMEM106A methylation and downregulation of protein expression. Receiver operating characteristic (ROC) curve analysis reveals that methylation of TMEM106A in tumor samples is different from that in non-malignant adjacent tissues of HCC patients. Moreover, HCC patients with TMEM106A hypermethylation have a poor clinical prognosis. 5-Aza-2′-deoxycytidin treatment of hypermethylated TMEM106A in highly metastatic HCC cells increases the expression of TMEM106A. Functional assays reveal that overexpression of TMEM106A significantly suppresses the malignant behavior of HCC cells *in vitro* and decreases tumorigenicity and lung metastasis *in vivo*. Mechanistically, TMEM106A inhibits epithelial mesenchymal transition (EMT) of HCC cells through inactivation of the Erk1/2/Slug signaling pathway. In conclusion, our findings demonstrate that TMEM106A is an inhibitor of HCC EMT and metastasis, and TMEM106A is often transcriptionally downregulated by promoter methylation, which results in reduced levels of TMEM106A protein and predicts poor survival outcomes for HCC patients.

## Introduction

Hepatocellular carcinoma (HCC) is a leading cause of cancer death worldwide [1]. Although surgical intervention remains the most common HCC treatment option, the prognosis for patients undergoing liver resection is still poor, with a high tumor recurrence rate. Therefore, understanding the molecular mechanisms that result in HCC metastasis is essential to improve patient outcomes.

Tumor occurrence and progression are typically considered to be driven by the accumulation of gene mutations [2]. However, this consideration excludes the potential influence of disrupted epigenetic mechanisms [ 3, 4] . Several studies have found that epigenetic modifications are involved not only in tumorigenesis but also in the regulation of the invasion and metastasis of tumor cells [ 5, 6] . DNA methylation, one of the most common epigenetic modifications, plays a critical role in transcriptional regulation through chromatin conformational modification and interference with transcription factor binding. Numerous tumor suppressor genes are frequently methylated in HCC, with methylation inactivation of tumor suppression, resulting in HCC dissemination. Hence, an understanding of the epigenetic mechanisms that underlie metastasis will promote discovery of predictors for HCC prognosis and the development of effective therapeutic treatments for HCC.

Epithelial mesenchymal transition (EMT), in which epithelial cells acquire mesenchymal features, has been implicated in numerous biological processes including stem cell biology, fibrosis/cirrhosis, and cancer [ 7, 8] . Our previous studies revealed a key role of EMT in HCC invasion and metastasis [ 9, 10] . Mechanistically, transcriptional repression of E-cadherin by multiple downstream transcription factors, such as Snail, Slug [11], DEF-1/ZEB-1 [12], SIP-1/ZEB-2 [13] and Twist [14], triggers EMT-mediated tumor progression. However, the involvement of the HCC upstream transcription factors is not well understood.

The gene for the transmembrane protein 106A (TMEM106A) is located in chromosomal region 17q21.31, encoding a novel protein with unknown function. Some evidence has implied that TMEM106A is involved in the suppression of tumor cell proliferation and migration by apoptosis induction [15]. Comprehensive and integrative genomic HCC studies have found that TMEM106A has high levels of tumor-specific methylation accompanied by decreased RNA expression [16]. These observations suggest that TMEM106A is a tumor suppressor gene that inhibits carcinogenesis and progression of HCC. However, the mechanistic basis for TMEM106A downregulation has not been elucidated, and a role for TMEM106A in HCC has not been clarified. Therefore, we analyze the mechanisms by which TMEM106A contributes to HCC metastasis and evaluate the potential clinical effectiveness of TMEM106A.

In the present study, we investigate TMEM106A gene expression and promoter methylation in human HCC cell lines in order to assess the relationship between promoter methylation and transcriptional inactivation of TMEM106A. In addition, we examine tumor-specific methylation and RNA expression of TMEM106A in a cohort of HCC patients with respect to clinico-pathologic patient characteristics and prognosis. Furthermore, we evaluate TMEM106A protein expression and gene methylation in a subset of HCC patients. Finally, the molecular mechanisms by which TMEM106A inhibits HCC tumor growth, invasion, and metastasis are explored by downregulation or overexpression of TMEM106A in HCC cell lines.

## Materials and Methods

### Cell culture and chemicals

HCCLM3, MHCC97-H, and MHCC97-L cells (human HCC cell lines with stepwise metastatic potential) were established at our lab [17]. Huh-7, PLC/PRF/5, and HepG2 cell lines (human HCC cells with poor metastatic potential) were obtained from the Cell Bank of the Chinese Academy of Sciences (Shanghai, China). All cell lines were routinely maintained and cultured in Dulbecco’s modified Eagle’s medium (DMEM; BD Biosciences, Franklin Lakes, USA), supplemented with 10% fetal bovine serum (BD Biosciences) in a humidified incubator containing 5% CO _2_ at 37°C. 5-Aza-2′-deoxycytidin was from Sigma (A3656; St Louis, USA). FR 180204, the Erk1/2 inhibitor, was purchased from Beyotime (SD5978; Shanghai, China).

### Lentivirusconstruction, cell transfection and clone selection

The hU6-MCS-Ubiquitin-EGFP-IRES-puromycin-shRNA-TMEM106Alentiviral vectors, the Ubi-MCS-3FLAG-SV40-EGFP-IRES-puromycin-TMEM106A cDNA lentiviral vectors, and the hU6-MCS-Ubiquitin-EGFP-IRES-puromycin-shRNA-Slug plasmid construction were purchased from Shanghai Genechem (Shanghai, China). The short hairpin RNA (shRNA) target sequences of TMEM106A and *Slug* are shown in Supplementary Table S1. The hU6-MCS-Ubiquitin-EGFP-IRES-puromycin-shRNA-mock lentiviral vectors and the Ubi-MCS-3FLAG-SV40-EGFP-IRES-puromycin-mock lentiviral vectors were used as controls. TMEM106A cDNA vectors were transfected into HCCLM3 and MHCC97H cells. shRNA-TMEM106A vectors were transfected into PLC/PRF/5 and HepG2 cells using Lipofectamine 2000 (Invitrogen, Carlsbad, USA) according to the manufacturer’s instructions. Transfected cells were selected under 10 μg/mL puromycin. Stably transfected clones were confirmed by quantitative real-time polymerase chain reaction (qRT-PCR) and western blot analysis.

### Patient and tissue specimens

Specimens were obtained from 332 HCC patients who received curative surgical resection in 2010 in Zhongshan Hospital. Of the 332 HCC patients, 60 paired tumor and peri-tumor snap-frozen tissues were used for qRT-PCR, DNA isolation, and methylation-specific PCR (MSP) analysis. An additional 30 paired tumor and peri-tumor paraffin-embedded tissues were collected for pyrosequencing and immunohistochemistry analysis to assess associations among TMEM106A methylation, protein expression, and patient prognosis. In addition, an additional 242 patients were randomly selected to construct tissue microarrays for the analysis of TMEM106A protein expression. These patients were monitored after surgery until July 7, 2015. The diagnosis for each patient was confirmed histopathologically. The patients did not exhibit signs of distant metastasis.

Tumors were assessed using the World Health Organization histological classification, with differentiation graded using the Edmondson-Steiner system. Liver function was assessed by the Child-Pugh scoring system. Tumor stage was determined according to the 2010 International Union Against Cancer tumor-node-metastasis classification system. Overall survival (OS) was determined as the time that elapsed between surgery and death or the last observation point. Data of surviving patients were censored at the last follow-up. Progress free survival (PFS) was defined as the interval between the date of surgery and the date of the first progression or the date of last follow-up. The protocols for the use of human subjects in this study were approved by the Research Ethics Committee of Zhongshan Hospital. Informed consents were obtained from all subjects.

### RNA isolation, reverse transcription and qRT-PCR

Total RNA was extracted from cell lines or tissues using Trizol reagent (T9424; Sigma). Complementary DNA synthesis was performed using High Capacity cDNA Reverse Transcription kit (4368813; Applied Biosystems, Foster City, USA) according to the manufacturer’s instructions. qRT-PCR was performed using SYBR™ Green PCR Master Mix (4309155; Applied Biosystems) to measure mRNA expression levels. Amplification and detection were performed using the Applied Biosystems Quant Studio 3 Digital PCR system. *GAPDH* was used as an endogenous control. Levels of target genes were calculated based on the cycle threshold (Ct) values and normalized to that of *GAPDH*, respectively, to yield a 2 ^–ΔCt^ value for relative expression of each transcript. The primers are shown in Supplementary Table S1. All experiments were performed in triplicate.

### Western blot analysis

Proteins were extracted using RIPA buffer (P0013B; Beyotime) supplemented with PMSF (ST505; Beyotime) and protease inhibitors (P1005; Beyotime). Then proteins were separated by 10% sodium dodecyl sulfate polyacrylamide gel electrophoresis, and then transferred to polyvinylidene difluoride membranes (Millipore, Billerica, USA). The membranes were washed and blocked with a buffer containing 5% BSA for 1 h at room temperature. The membranes were incubated with the primary antibodies at 4°C overnight, followed by incubation with the corresponding horseradish peroxidase-conjugated secondary antibodies for 1 h at room temperature. Finally, protein bands were detected using an enhanced chemiluminescence kit (Beyotime). Tubulin was used as the loading control. The antibodies used are listed in Supplementary Table S2.

### Immunofluorescence microscopy

Cells were cultured on glass slides and fixed with 4% paraformaldehyde for 15 min. The fixed cells were then permeabilized with 0.1% Triton X-100 for 15 min at room temperature, washed with phosphate-buffered saline (PBS) and blocked with PBS containing 1% (w/v) bovine serum albumin (BSA) and 0.15% (w/v) glycine for 1 h at room temperature. Then cells were incubated with primary antibody for 2 h at room temperature. A negative control (without primary antibody) was included on each slide. Cells were washed and incubated with HRP-conjugated secondary antibody for 1 h at room temperature. After rinsing with PBS, the slides were counterstained with diamidinophenylindole (DAPI) and examined under a fluorescence microscope (Leica Microsystems Imaging Solutions, Cambridge, UK).

### Tissue microarray and immunohistochemistry

The construction of tissue microarray (TMA) and the immunohistochemistry analysis were performed as described previously [18]. Formalin-fixed paraffin-embedded tissue sections were stained for TMEM106A using the horseradish peroxidase immunohistochemistry kit (Gene Tech, Shanghai, China) according to the manufacturer’s instructions. Briefly, after rehydration and microwave antigen retrieval, monoclonal rabbit anti-TMEM106A antibody (1:150 dilution; Abcam) were applied to the slides, and incubated at 4°C overnight. Then, the slides were incubated with HRP-conjugated secondary antibody (Gene Tech) at 37°C for half an hour. Staining was developed using diaminobenzidine (DAB), and Mayer′s hematoxylin was used for counterstaining. Negative control slides were probed with bovine serum albumin (BSA) under the same experimental conditions. Images of representative fields were captured using a microscope (Leica Microsystems Imaging Solutions) and analyzed using Leica QWin Plus v3 software. For comparison with methylation results and survival analysis, TMEM106A positivity was defined when scored as moderate staining (2+) or strong staining (3+), while TMEM106A negativity was defined when scored as no staining (0) or weak staining (1+).

### DNA isolation and MSP analysis

Genomic DNA was extracted from HCC cell lines and tissue specimens using the proteinase-K method. Modified EpiTect Fast Bisulfite Conversion kits (Qiagen, Hilden, Germany) were used according to the manufacturer’s instructions. The MSP primers used for TMEM106A methylation analysis are listed in Supplementary Table S1 and synthesized according to genomic sequences flanking the presumed transcription start sites. The MSP reaction was carried out as previously described [19].

### Bisulphite genomic sequencing (BGS) and pyrosequencing

Genomic DNA from HCC cell lines was denatured and converted with sodium bisulphite using EZ DNA Methylation-Gold Kit ^TM^ (D5005; Zymo Research, Orange, USA) according to the manufacturer’s instructions. The primers used to amplify the promoter regions of TMEM106A are shown in Supplementary Table S1. The amplified DNA products were integrated into a pMD19-T vector and transformed into *Escherichia coli* according to the manufacturer’s protocol. Five colonies were selected for subsequent sequencing for methylation detection. Additionally, genomic DNA from HCC tissue samples was also used for pyrosequencing. The promoter regions of TMEM106A were amplified and purified to single-stranded DNA as the template for the pyrosequencing reaction performed with PyroMark Gold Q96 (Qiagen). The pyrosequencing primers shown in Supplementary Table S1 were used to analyze the sequence of YGTTTTYGTATTTTYGATTTTATTTTTTTTYGTTTTTGTYGTTG.

### Cell proliferation assay

Cell proliferation was measured by CCK-8 assay kit according to the manufacturer’s protocols. Briefly, cells (2000 cells/well) were dispensed in 100-μL aliquots into a 96-well plate and cultured. At the indicated time points, 10 μL CCK-8 solution (Dojindo, Tokyo, Japan) was added to the cells, and plates were incubated for another 2 h. The absorbance at 450 nm was measured to determine the number of viable cells in each well.

### Wound healing assay

Cell migration was evaluated by scratch wound healing assay. Cells were cultured for 2 days to form a tight cell monolayer and then serum-starved for 16 h. After serum starvation, the cell monolayer was wounded with a 10-μL plastic pipette tip. The remaining cells were washed twice with culture medium to remove cell debris and incubated at 37°C with normal serum-containing culture medium. At the indicated time points, migrating cells at the wound front were photographed using an inverted microscope (OLYMPUS, Tokyo, Japan). The percentage of the cleared area at each time point compared with time zero was measured using Image-Pro Plus v6.2 software.

### Matrigel transwell assays

Cell invasion was determined using 24-well transwells (8-μm pore size; Corning, New York, USA) precoated with Matrigel (BD Biosciences). A total of 1×10 ^5^ cells were suspended in 100 μL Dulbecco’s modified Eagle medium (DMEM; BD Biosciences) with 1% fetal bovine serum and were added to the upper chamber, and 600 μL DMEM with 10% fetal bovine serum was added in the lower chamber. After 48 h of incubation, the cells remaining in the upper chamber were removed using cotton swabs. Cells on the lower surface of the membrane were fixed in 4% paraformaldehyde and stained with Giemsa. Cells in five microscopic fields were photographed at 200× magnification and counted.

### *In vivo* mouse tumorigenicity and metastasis assays

Four-to-six-week old male BALB/c *nu*/ *nu* mice were obtained from Shanghai Institute of Materia Medica, the Chinese Academy of Sciences (Shanghai, China) and housed under specific-pathogen-free conditions. Humane animal care protocols were conducted as described by the National Research Council Guide for the Care and Use of Laboratory Animals.

HCC cells (5×10 ^6^) were suspended in 100 μL serum-free DMEM (1:1; BD Biosciences) and were injected subcutaneously into the flank region of each immune deficient BALB/C nude mice. When the subcutaneous tumor reached approximately 1 cm ^3^ in volume (approximately 5 weeks after injection), it was removed, minced into small pieces of equal volume (2 ×2×2 mm ^3^), and transplanted into the livers of nude mice (6 in each group). All mice were monitored once every 5 days and sacrificed 6 weeks post-inoculation. The weight of each tumor was measured. The tumor volume (mm ^3^) was calculated as follows: *V* = *ab* ^2^⁄2, where *a* and *b* are the largest and smallest tumor diameters measured at necropsy, respectively. The orthotopic tumors in a potentially involved metastatic organ, lung, were harvested, weighed, fixed, and embedded in paraffin, and the total number of lung metastases was counted under a microscope as described previously [20]. Tumor tissue sections were prepared and immunoreactivity was analyzed as previously described, using anti-TMEM106A antibody and anti-Ki67 antibody.

### Gene set enrichment analysis (GSEA)

Gene expression profile data for HCC was downloaded from The Cancer Genome Atlas (TCGA). GSEA 4.0.2 version software ( http://software.broadinstitute.org/gsea/datasets.jsp) was used to analyze the edited TCGA data and gene sets with MsigDB (Molecule Signatures Database) serving as a reference gene set [ 21, 22] . Each analysis was repeated 1000 times. The False Discovery Rate (FDR), Enrichment Score (ES), Normalized Enrichment Score (NES), and *P*-value were included in the GSEA.

### Statistical analysis

Quantitative data between groups were compared using the Student’s *t* test or Mann-Whitney U test. Categorical data were analyzed by the χ ^2^ test or Fisher exact test. A Cox proportional hazards regression model was applied to perform univariate and multivariate analyses. Those variables that achieved statistical significance in the univariate analysis were entered into multivariable analysis. For methylation analysis, TMEM106A methylation in the tumor and in the peri-tumor tissues was used to calculate a methylation signal ratio (with a ratio of >1.5 considered methylation positive). The Kaplan-Meier method with the log-rank test was used to analyze the influence of TMEM106A protein expression or DNA methylation on progression-free survival (PFS) and overall survival (OS). Statistical analysis was performed with the Statistical Package for Social Sciences version 24.0 (SPSS Inc., Chicago, USA) and Graphpad software (San Diego, USA). *P*<0.05 was considered statistically significant.

## Results

### Transcriptional silencing of TMEM106A in HCC cells is due to methylation

**Figure 1:**
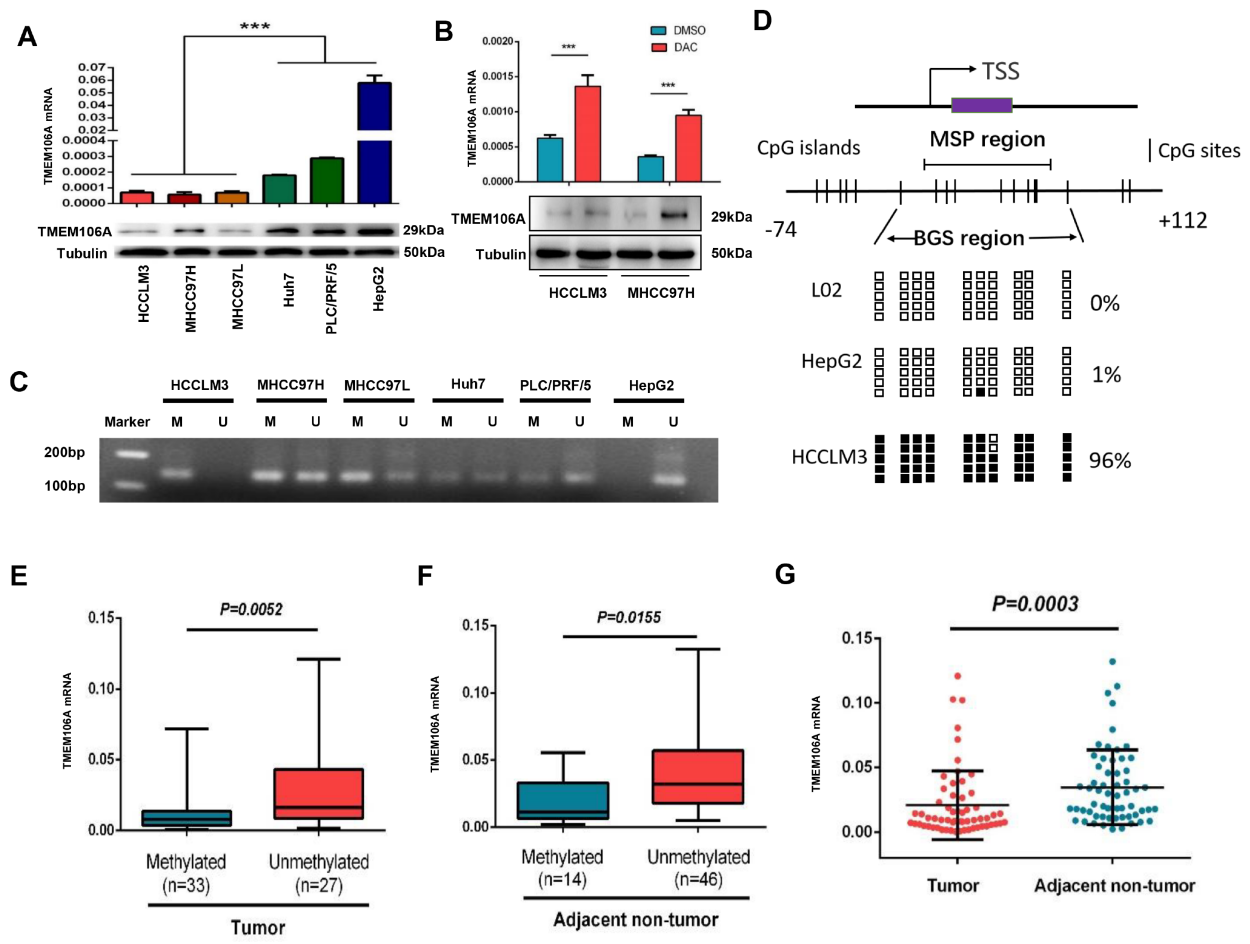
Figure 1 TMEM106A is downregulated by promoter region methylation in HCC (A) Expression of TMEM106A in HCC cell lines with different metastatic potential. (B) Expression of TMEM106A in HCCLM3 and MHCC97H after treatment with Decitabine (DAC). (C) MSP was used to evaluate the methylation of the TMEM106A promoter region in different HCC cell lines. M, methylated; U, unmethylated. (D) CpG island mapping of the promoter region of TMEM106A based on BGS analysis of the human hepatic cell line L02 and HCC cell lines (HepG2 and HCCLM3 with low and high metastatic potentials). TSS, transcription start site; black squares indicate methylated CpG sites, white squares indicate unmethylatedCpG sites. (E) qRT-PCR measurement of the expression of TMEM106A in tumor tissues with methylated or unmethylated promoter regions. (F) The expression of TMEM106A in adjacent non-tumor tissues with methylated or unmethylated promoter regions. (G) TMEM106A expression in HCC tissues was significantly lower than that in adjacent noncancerous tissues ( n=60). Data are shown as the mean±SD. *** P<0.001.

TMEM106A expression in various human HCC cell lines was examined by qRT-PCR and western blot analysis. TMEM106A expression was increased at the mRNA and protein levels in the poorly metastatic HCC cell lines (PLC/PRF/5, Hep G2, and Huh7), compared with that in the highly metastatic HCC cell lines (MHCC97L, MHCC97H, and HCCLM3; *P*<0.001; Figure 1A). These results suggest a negative relationship between TMEM106A expression and the metastatic potential of HCC cells. MSP was used to determine whether DNA methylation of the TMEM106A promoter results in reduced expression of TMEM106A in highly metastatic HCC cells. TMEM106A was found to be hypermethylated in highly metastatic HCC cells, with promoter hypomethylation in poorly metastatic HCC cells ( Figure 1C). 5-Aza-2′-deoxycytidin treatment of hypermethylated TMEM106A in HCCLM3 and MHCC97H cells increased the expression of TMEM106A compared with that in untreated HCC cells ( Figure 1B). The 5′-regions of TMEM106A in HCC cell lines (Hep G2 and HCCLM3) and the human hepatic cell line L02 were assessed by BGS. Our results demonstrated that 96% of CpG sites of TMEM106A were methylated in HCCLM3 cells. Only 1% of TMEM106A CpG sites were methylated in HepG2 and no CpG site was methylated in L02 cells ( Figure 1D).

### TMEM106A is specifically methylated in tumor tissues of HCC patients

**Figure 2:**
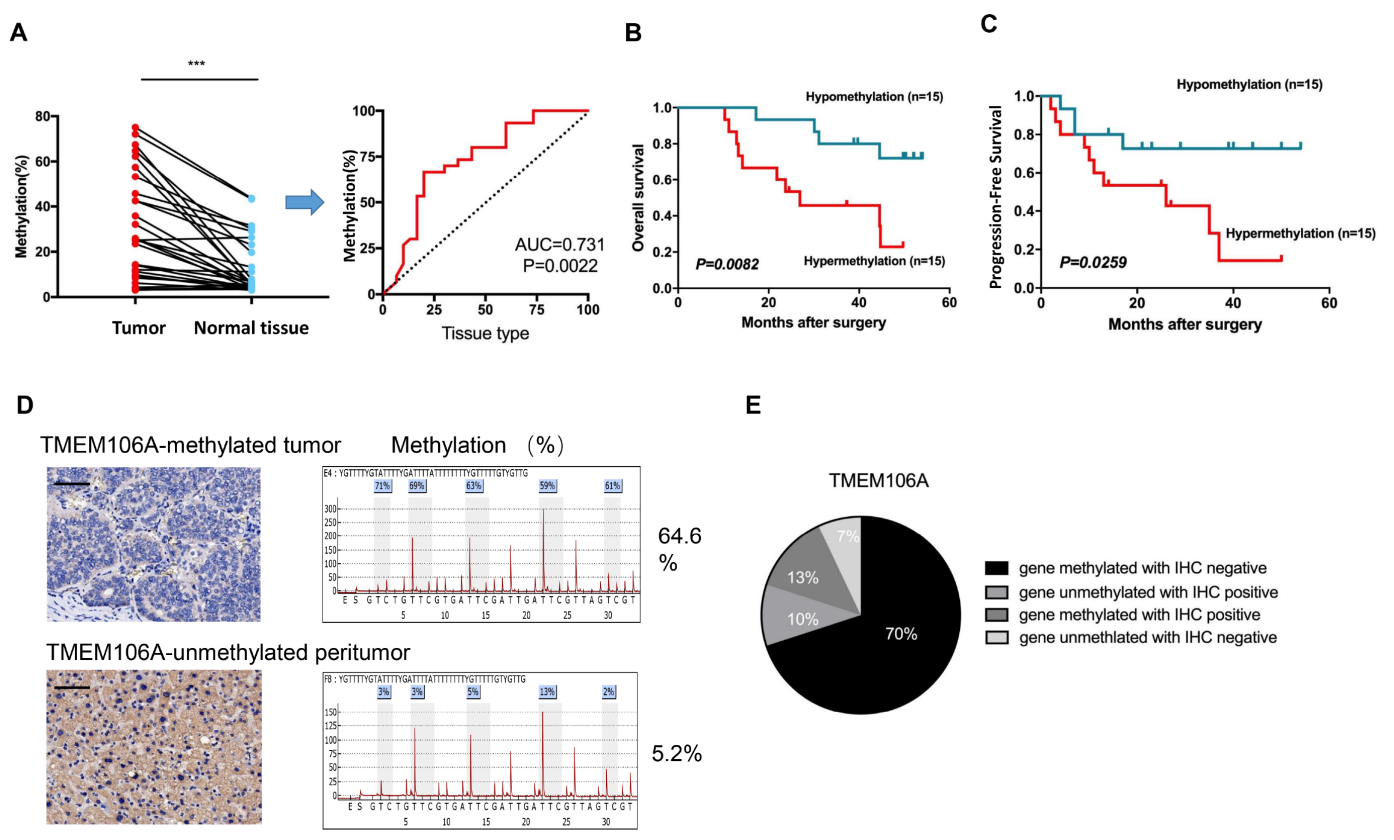
Figure 2 TMEM106A is specifically methylated in HCC patients and is associated with a poor prognosis (A) Statistically significant comparison of TMEM106A methylation percentage in tumors and normal tissues from 30 paired HCC patients. Analysis of the ROC curve showed that the level of TMEM106A methylation can distinguish tumor tissues from normal tissues. Red line, methylation of TMEM106A; AUC, area under the curve. (B) Kaplan-Meier analyses of overall survival (OS) rate based on TMEM106A methylation status ( n=30). (C) Kaplan-Meier analyses of progress-free survival (PFS) rate based on TMEM106A methylation status ( n=30). (D) Representative immunohistochemical staining of TMEM106A and paired methylation percentage in tumor and peri-tumor tissues. In TMEM106A-methylated tumor tissues, TMEM106A expression was not observed. In TMEM106A-unmethylated peri-tumor tissues, cytoplasmic expression of TMEM106A was observed in HCC cells. Scale bar, 100 μm. (E) Representative comparison of TMEM106A protein expression and TMEM106A methylation status in HCC tissues ( n=30). IHC, immunohistochemistry. *** P<0.001.

TMEM106A methylation and mRNA expression were examined in tumors and corresponding paired peri-tumor tissues of 60 HCC patients. MSP assays showed that TMEM106A was methylated in 55% (33/60) of tumor tissues and 23.3% (14/60) of peri-tumor tissues ( *P*<0.001; Figure 1E,F). TMEM106A mRNA expression in tumor tissues was significantly decreased compared with that in peri-tumor tissues ( Figure 1G). These results demonstrate that low TMEM106A mRNA expression is significantly related to hypermethylation of TMEM106A in HCC tissues. To assess the clinical significance of TMEM106A methylation, DNA pyrosequencing analysis of TMEM106A was performed in another cohort of 30 HCC patients. By comparison of the ratio of paired tumor to peri-tumor tissue, TMEM106A had a greater level of methylation in HCC tumor tissues of 83% of patients (ratio≥1.5) [23]. Receiver operating characteristic (ROC) curve analysis revealed TMEM106A methylation in tumor samples was different from that in non-malignantadjacent tissues of HCC patients ( *P*<0.01; AUC=0.731; Figure 2A).

Moreover, using a median cutoff for TMEM106A methylation in tumors (higher than median level=hypermethylation, and lower than median level=hypomethylation), the 5-year OS of TMEM106A-hypermethylated HCC patients was significantly lower than that of the TMEM106A-hypomethylated patients ( *P*=0.0082; Figure 2B). Similarly, HCC patients with TMEM106A hypermethylation had a lower PFS than patients with TMEM106A hypomethylation ( *P*=0.0259; Figure 2C). Furthermore, TMEM106A methylation was significantly correlated with tumor size ( *P*=0.008). Other clinico-pathological parameters such as age, tumor number, preoperative serum alpha-fetoprotein, vascular invasion, and tumor differentiation were not correlated with TMEM106A methylation ( Supplementary Table S3).

### Loss of TMEM106A protein expression in HCC patients predicts a poor prognosis

**Figure 3:**
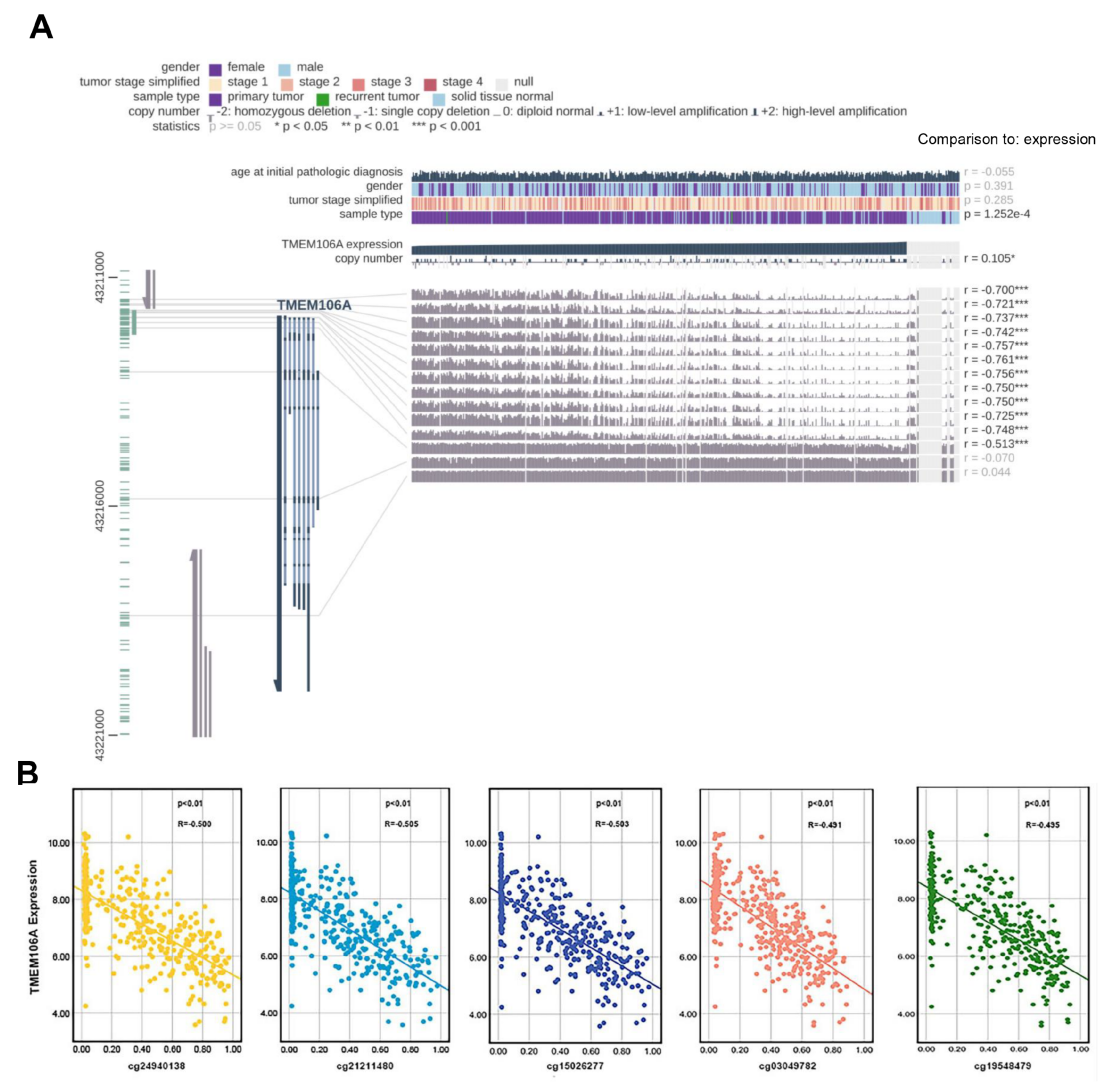
Figure 3 Analysis of the association between DNA methylation and TMEM106A dysregulation (A) Analysis of the relationship between TMEM106A expression and TMEM106A CpG island methylation in TCGA database using the MEXPRESS tool. Pearson’s correlation coefficients (r) of methylation sites are shown on the right side. (B) The negative correlation between TMEM106A expression and TMEM106A CpG island methylation was shown. TCGA, The Cancer Genome Atlas. *** P<0.001.

To investigate the relationship between TMEM106A methylation and TMEM106A protein level, tumors and peri-tumor tissues of 30 HCC patients were assessed by immunohistochemistry and pyrosequencing. TMEM106A protein expression was undetectable in 23% of patients, with low levels of staining in 53% of the tumor tissues. Moderate or intense TMEM106A staining was observed in 17% and 7% of the tumor tissues, respectively. Typical immunohistochemistry staining of tumors and peri-tumor tissues with corresponding methylation status are shown in Figure 2D. Comparison of TMEM106A methylation and TMEM106A protein expression in tumor tissues revealed downregulation of TMEM106A protein expression in 70% of TMEM106A-methylated tumor tissues ( Figure 2E). The status of 14 CpG sites of 468 HCC samples in the TCGA database was assessed using MEXPRESS [24]. Twelve CpG sites were negatively correlated with TMEM106A expression, and another two sites were not associated with TMEM106A expression ( Figure 3A). As shown in Figure 3B, the Pearson negative correlations exist between TMEM106A expression and the twelve CpG sites including cg19548479, cg24940138, cg21211480, cg15026277, cg03049782, cg24008544, cg18222083, cg04482110, cg25918947, cg21504064, cg04524477, and cg08377924. Collectively, these results suggest a possible role for DNA methylation in regulation of the abnormal expression of TMEM106A in HCC.

**Figure 4:**
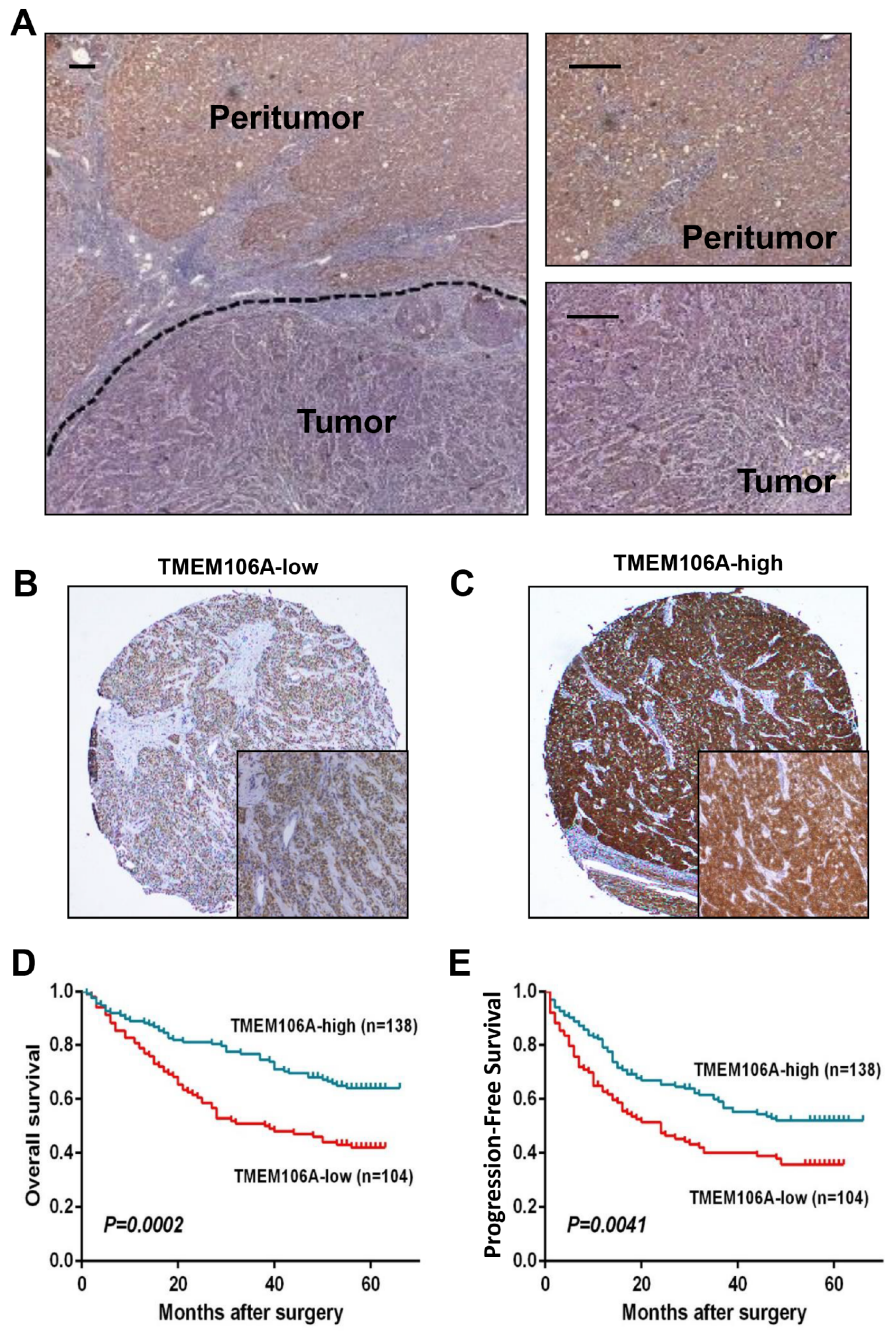
Figure 4 Downregulation of TMEM106A expression predicts a poor prognosis for HCC patients (A) Expression of TMEM106A in peri-tumor and tumor tissues detected by immunohistochemical staining. (B) Representative immunohistochemical staining images with low expression of TMEM106A in tissue microarrays. Scale bar, 100 μm. (C) Representative immunohistochemical staining images with high expression of TMEM106A in tissue microarrays. Scale bar, 100 μm. (D) Kaplan-Meier analyses of OS rate based on TMEM106A expression ( n=242). (E) Kaplan-Meier analyses of PFS rate based on TMEM106A expression ( n=242).

To evaluate TMEM106A expression as a prognostic predictor for HCC patients after surgical resection, TMEM106A expression was assessed by a tissue microarray composed of a cohort of 242HCC patients. The clinical parameters of these patients aresummarized in Supplementary Table S4, with representative immunohistochemistry staining of TMEM106A in Figure 4A–C. The 1-, 3-, and 5-year OS rates of the TMEM106A ^Low^ group (78.8%, 51.0% and 42.0%, respectively) were significantly lower than the OS rates of the TMEM106A ^High^ group (89.1%, 76.8% and 64.1%, respectively; Figure 4D). Likewise, the TMEM106A ^Low^ group had lower PFS rate than the TMEM106A ^High^ group (1-year PFS, 62.8% versus 79.2%; 3-year PFS, 40.0% versus 59.1%; and 5-year PFS, 35.8% versus 52.0%; Figure 4E). Multivariate analysis indicated that TMEM106A expression is an independent predictor of OS and PFS (hazard ratio 0.541, *P*<0.01; and hazard ratio 0.658, respectively, *P*<0.05; Supplementary Table S5), as well as tumor size, tumor number, and tumor differentiation.

### Effects of TMEM106A on HCC growth and metastasis

**Figure 5:**
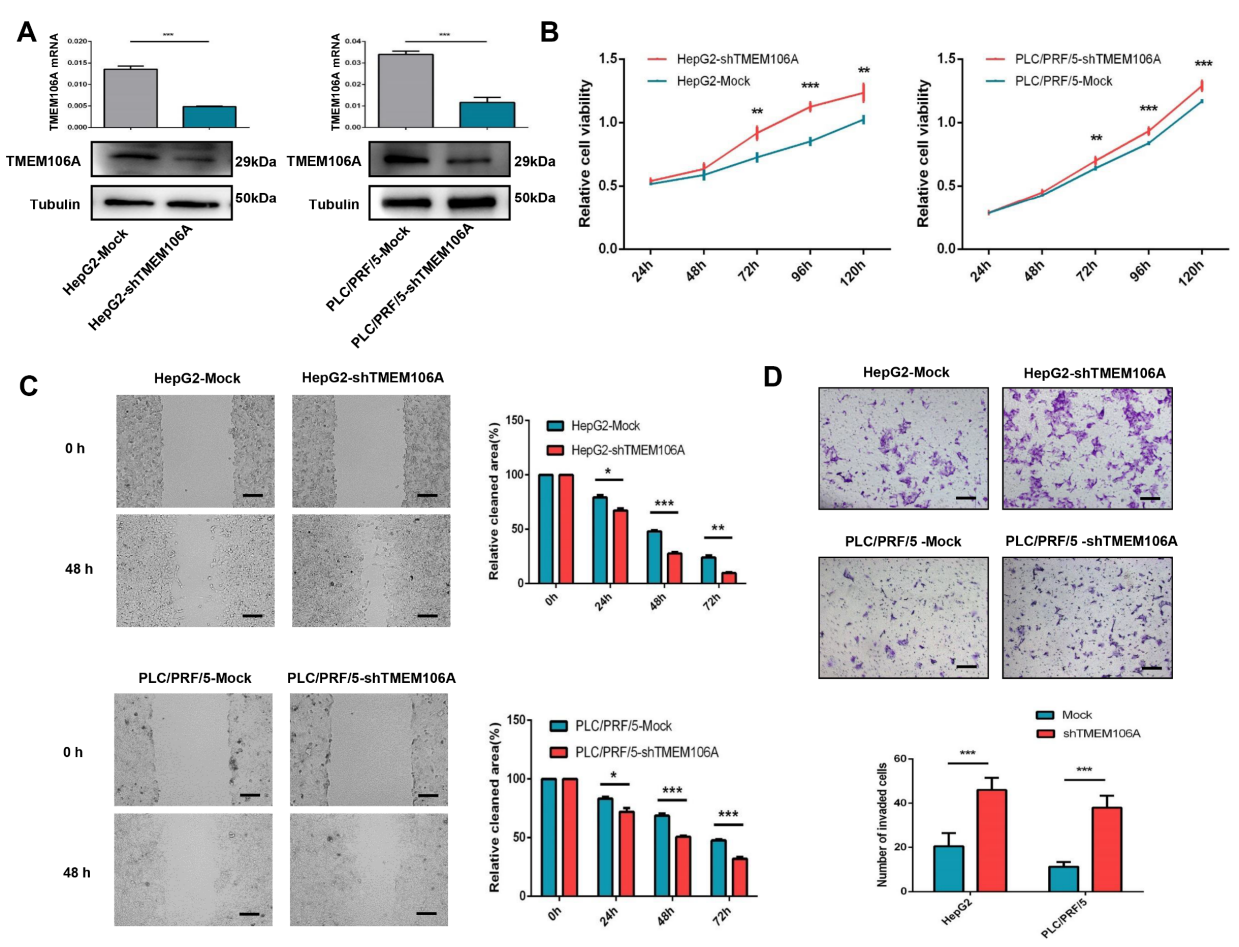
Figure 5 **TMEM106A knockdown promotes proliferation, migration, and invasion of HCC cells** *in vitro* (A) qRT-PCR and western blot analysis showed knockdown of TMEM106A in HepG2 and PLC/PRF/5 cells after transfection with shRNA-TMEM106A. (B) Growth curves of the indicated cells in cell proliferation assays. (C) Wound-healing migration assays and the quantification of relative clear area. (D) Representative images and quantification of tumor cell invasion in Matrigel invasion assays. Scale bar, 100 μm. Data are shown as the mean±SD. * P<0.05, ** P<0.01, and *** P<0.001.

To explore the role of TMEM106A in HCC progression *in vitro* and *in vivo*, we established stable TMEM106A-knockdown Hep G2 and PLC/PRF/5 cells ( Figure 5A), as well as stable TMEM106A-overexpressing MHCC97H and HCCLM3 cells ( Supplementary Figure S1A). *In vitro*, enhanced proliferative capacity was observed after downregulation of TMEM106A expression in PLC/PRF/5 and Hep G2 cells ( Figure 5B). Cell proliferation was reduced with TMEM106A up-regulation in MHCC97H and HCCLM3 cells ( Supplementary Figure S1B). Wound healing assay showed that HepG2-shTMEM106A and PLC/PRF/5-shTMEM106A cells exhibited significantly increased rates of wound closure when compared with the corresponding control cells ( Figure 5C). Meanwhile, HCCLM3-TMEM106A and MHCC97H-TMEM106A cells had significantly delayed rates of wound closure compared with the corresponding controls ( Supplementary Figure S1C). Matrigel invasion assay demonstrated that HepG2-shTMEM106A and PLC/PRF/5-shTMEM106A cells had increased invasive capacity compared with the corresponding control cells (46.0±5.5 vs 20.5±6.0, *P*=0.001; 38.0±5.4 vs 11.3±2.2, *P*=0.001, respectively; Figure 5D). Likewise, decreased invasiveness was observed in MHCC97H-TMEM106A and HCCLM3-TMEM106A cells when compared with their corresponding control cells (14.0±2.2 vs 46.3±5.9, *P*=0.001; 28.3±11.7 vs 67.3±8.0, *P*=0.002; Supplementary Figure S1D).

**Figure 6:**
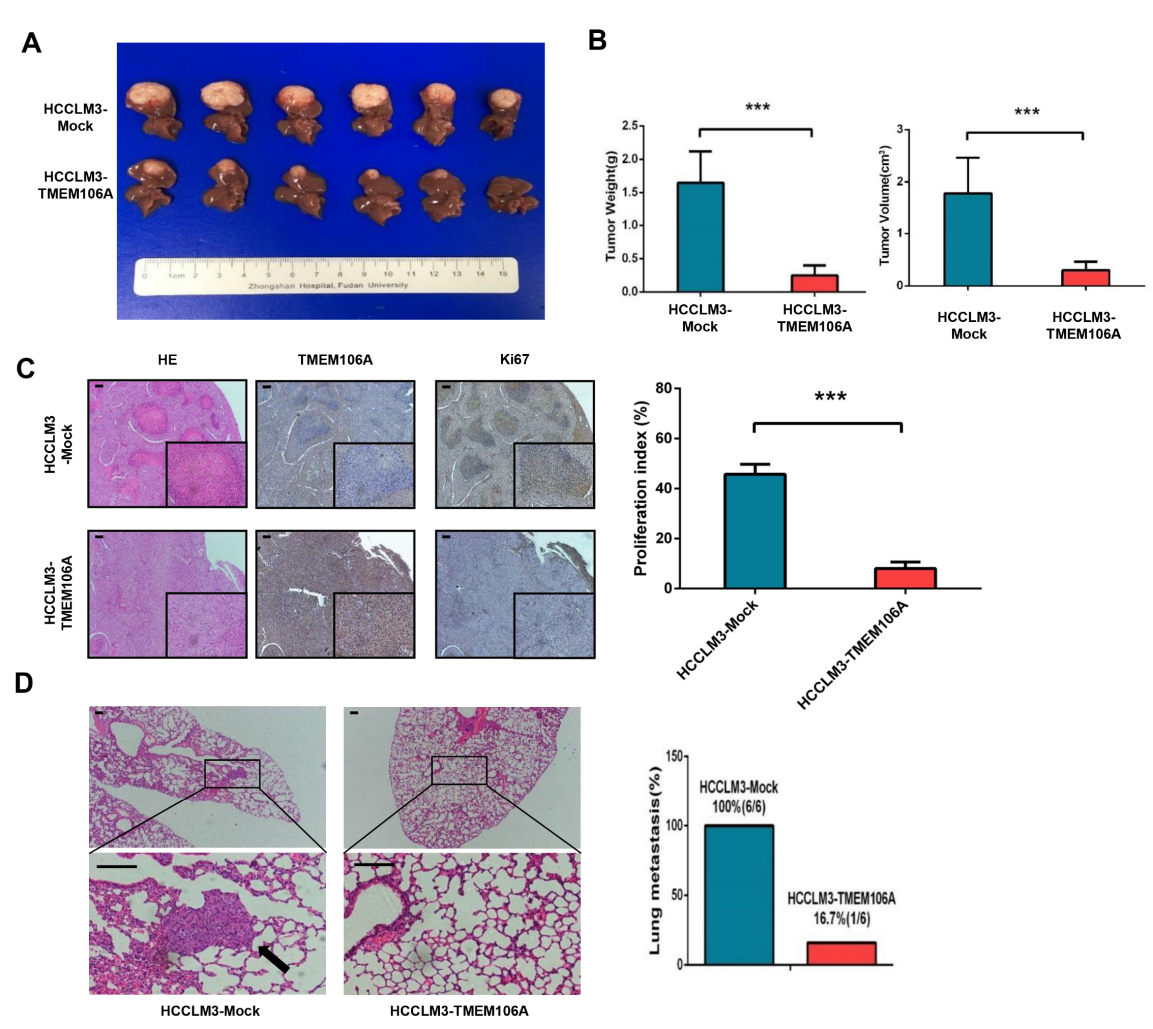
Figure 6 **TMEM106A overexpression inhibits tumorigenesis and lung metastasis of HCC cells** ***in** ***vivo**** (A) Tumors derived from the HCCLM3-Mock group and HCCLM3-TMEM106A group. (B) Weight and volume of tumors derived from the HCCLM3-Mock group and HCCLM3-TMEM106A group. (C) Immunohistochemical staining images indicating the expressions of TMEM106A and Ki67 in tumors derived from the HCCLM3-Mock group and HCCLM3-TMEM106A group. (E) Representative images of lung tissue sections from each group and the corresponding pulmonary metastasis rate. Scale bar, 100 μm. Data are shown as the mean±SD. *** P<0.001.

Since overexpression of TMEM106A in HCC cells significantly inhibited cell migration and invasion, we established a tumor xenograft model to explore the effect of TMEM106A on HCC growth and metastasis *in vivo*. It was found that tumor size and tumor weight of HCCLM3-Mock-derived xenografts were 1.8± 0.3 cm ^3^ and 1.6± 0.2 g respectively, which were significantly larger than those of HCCLM3-TMEM106A-derived xenografts (0.3± 0.1 cm ^3^, *P*<0.001; 0.2± 0.1 g, *P*<0.001, respectively; Figure 6A,B). Moreover, the HCCLM3-Mock cells exhibited a significantly increased proliferation rate when compared with HCCLM3-TMEM106A cells ( *P*<0.001; Figure 6C). The incidence of lung metastasis of HCCLM3-Mock cells was 100% (6/6), which was significantly higher than that ofHCCLM3-TMEM106A cells (1/6) ( Figure 6D). These results demonstrate that TMEM106A acts as a tumor suppressor during HCC progression *in vivo*.

### TMEM106A knockdown promotes EMT through upregulation of Slug expression

**Figure 7:**
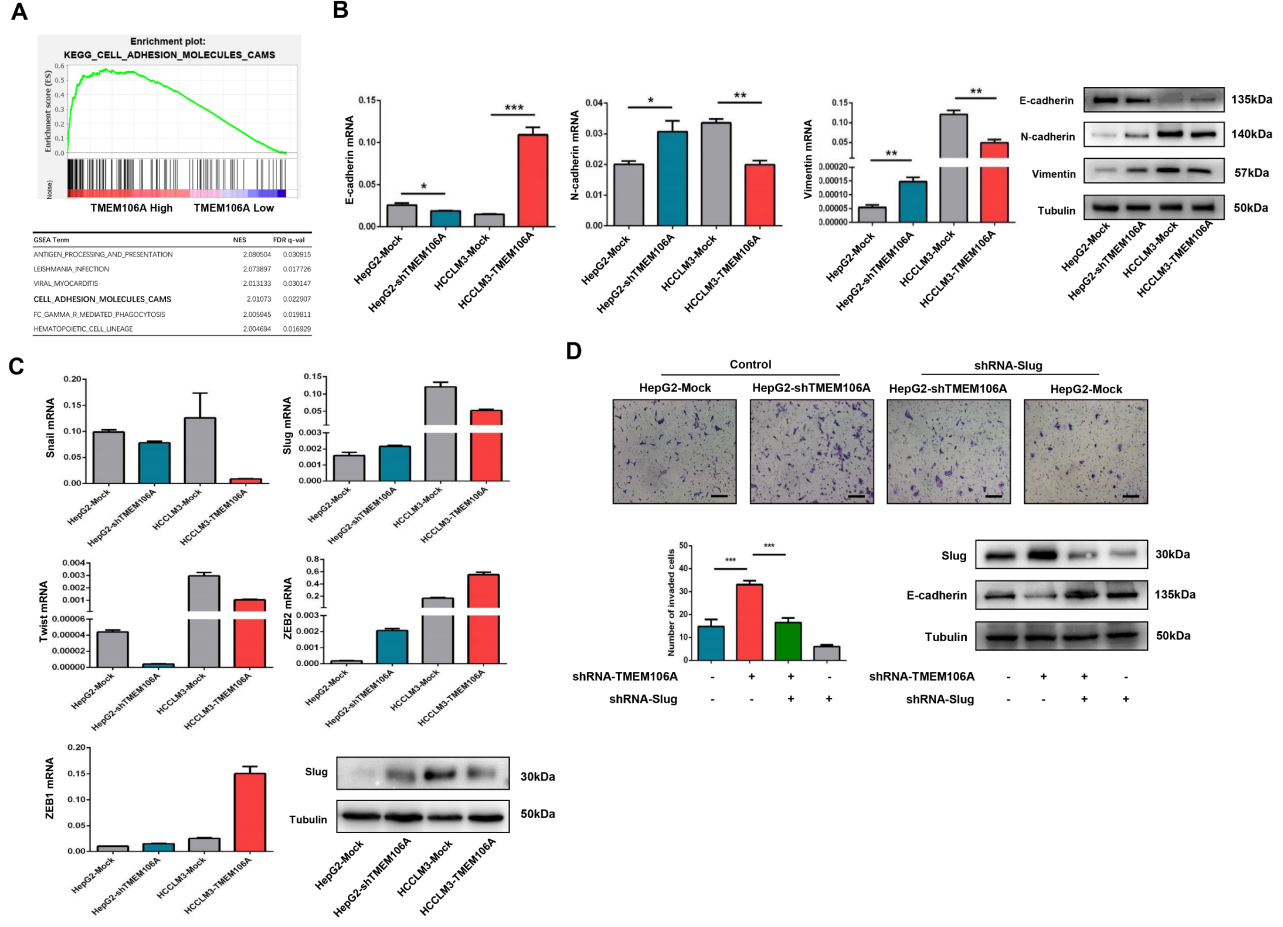
Figure 7 TMEM106A knockdown induces EMT by regulating Slug in HCC (A) GSEA plot demonstrating enrichment of genes in the cell adhesion molecule pathway for HCC patients with high TMEM106A expression. (B) Changes in expression of EMT markers following stable up- or downregulation of TMEM106A expression. (C) Changes in expression of EMT-transcription factors following stable up- or downregulation of TMEM106A expression. (D) Invasive ability and expression of E-cadherin in HepG2-Mock and HepG2-shTMEM106A after transfection with shRNA specific for Slug. shRNA, short hairpin RNA. Scale bar, 100 μm. Data are shown as the mean±SD. * P<0.05, ** P<0.01, and *** P<0.001.

To understand why TMEM106A inhibits tumor progression, GSEA analysis was performed using the TCGA database. TMEM106A was found to be positively related to cell adhesion pathways ( Figure 7A), suggesting a role of TMEM106A in maintaining the epithelial phenotype and inhibiting HCC EMT. To validate the role of TMEM106A in EMT, the expressions of three key EMT biomarkers were assessed by qRT-PCR and western blot analysis, after downregulation of TMEM106A expression in HepG2 cells and overexpression of TMEM106A in HCCLM3 cells. Results showed that E-cadherin expression was inhibited in HepG2-shTMEM106A cells, while N-cadherin and Vimentin expressions were upregulated ( Figure 7B). Overexpression of TMEM106A increased E-cadherin expression and decreased N-cadherin and Vimentin expressions in HCCLM3-TMEM106A cells ( Figure 7B).

Several transcription factors including Snail, Slug, Twist, ZEB1, and ZEB2 often participate in the regulation of EMT. Therefore, we examined their expressions in HepG2-shTMEM106A and HCCLM3-TMEM106A cells by qRT-PCR. As shown in Figure 7C, Slug expression was increased after downregulation of TMEM106A expression in HepG2 cells. TMEM106A overexpression in HCCLM3 cells decreased Slug expression, whereas the expressions of Snail, Twist, ZEB1, and ZEB2 were not markedly changed. In order to confirm the function of Slug in TMEM106-regulated EMT and HCC invasion, shRNAs for Slug were transfected into HepG2-Mock and HepG2-shTMEM106A cells. Inhibition of Slug in HepG2-shTMEM106A cells increased the expression of E-cadherin compared with that inHepG2-shTMEM106A cells. Enhanced invasion capacity induced by TMEM106A knockdown was significantly inhibited by Slug shRNA ( Figure 7D). These results suggest a critical role of Slug in TMEM106-regulated EMT and HCC invasion.

### Erk1/2/Slug signaling plays a critical role in EMT of HCC cells induced by TMEM106A knockdown

**Figure 8:**
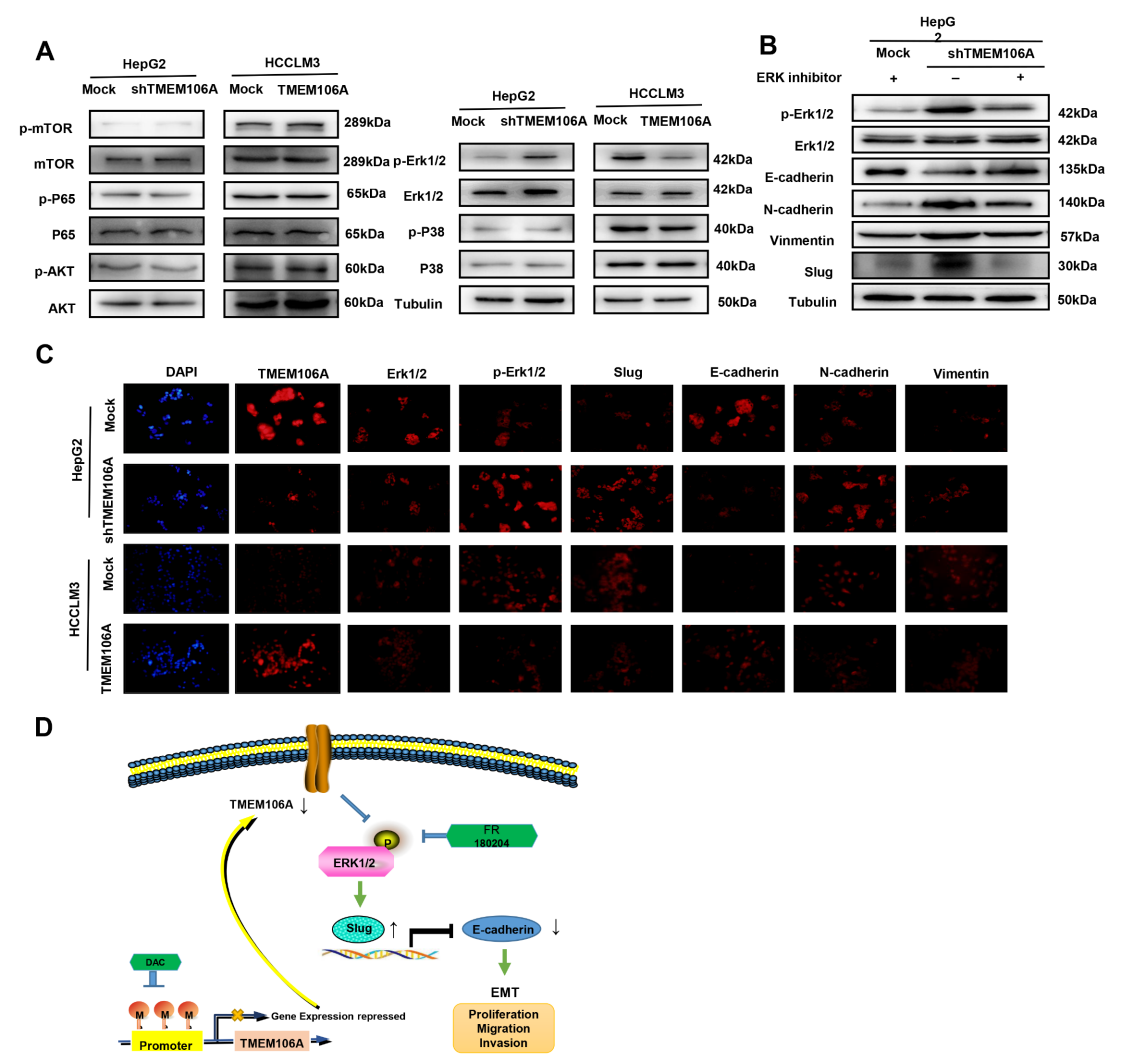
Figure 8 ***TMEM106A*** **knockdown induces EMT through activating the Erk1/2/Slug signaling pathway** (A) Western blot analysis was used to detect the phosphorylation of common EMT-related signaling pathways in HepG2-Mock, HepG2-shTMEM106A, HCCLM3-Mock, and HCCLM3-TMEM106 cells. (B) Erk1/2 inhibitor partially reverses EMT of HCC cells induced by TMEM106A knockdown. (C) Immunofluorescence staining analysis verified that TMEM106A suppressed invasion and metastasis of HCC through the Erk1/2/Slug signaling pathway. Scale bar, 100 μm. (D) Schematic diagram of downregulation of TMEM106A protein expression by hypermethylated TMEM106A promoter, resulting in EMT, tumor cell invasion, and metastasis of HCC.

Growing evidence has shown that EMT can be induced in the context of tumorigenesis by several cancer-associated signaling cascades. These include NF-κB pathway, mTOR pathway, phosphoinositide 3-kinase (PI3K)/AKT pathway, MAPK pathway, and Erk1/2-dependent pathway [ 25– 27] . To explore the signaling pathways by which TMEM106A regulates Slug and inhibits HCC EMT, we examined phosphorylation levels of these pathways by western blot analysis of HepG2-shTMEM106A and HCCLM3-TMEM106A cells. The results showed that inhibition of TMEM106A in HepG2 cells significantly increased Erk1/2 phosphorylation compared with that in HepG2-Mock cells. Furthermore, upregulation of TMEM106A expression in HCCLM3 cells significantly decreased the phosphorylation levels of Erk1/2, but had no impact on mTOR, P65, P38, or AKT phosphorylation ( Figure 8A). To determine whether TMEM106A knockdown induces HCC EMT by activating the Erk1/2/Slug signaling pathway, HepG2-shTMEM106A cells were treated with an Erkl/2 inhibitor, FR 180204. Results showed that the Erk1/2 inhibitor partially reversed HCC EMT induced by TMEM106A knockdown ( Figure 8B). Inhibition of Erkl/2 in HepG2-shTMEM106A cells also decreased Slug expression compared with that in HepG2-shTMEM106A cells, despite low TMEM106A level. Immunofluorescence staining assay confirmed that downregulation of TMEM106A expression promoted HCC EMT by activation of the Erk1/2/Slug signaling pathway ( Figure 8C).

## Discussion

Emerging evidence has demonstrated that the TMEM family, including TMEM176A and TMEM45B, is involved in tumor progression, metastasis, and chemo-resistance. These family members function as tumor suppressors or oncogenes [ 28– 30] . As a member of this family, TMEM106A is a type II transmembrane protein with poorly characterized function. Previous studies have reported the downregulation of TMEM106A expression during tumorigenesis of lung and breast cancers [ 15, 31] . However, the role of TMEM106A in HCC occurrence and progression is largely unknown. Herein, TMEM106A was demonstrated to have lower expression in highly metastatic hepatoma cells compared with that in poorly metastatic hepatoma cells. Low TMEM106A protein level in HCC tissues was associated with poor patient survival after surgical resection. Furthermore, TMEM106A expression was found to be an independent prognostic predictor for OS and PFS, as judged by univariate and multivariate analysis. *In vitro* and *in vivo* functional assays demonstrated that TMEM106A inhibit migration, invasion, and lung metastasis of HCC cells. Taken together, these results demonstrate that TMEM106A is a novel anti-oncogene involved in the inhibition of tumor growth and progression. To the best of our knowledge, this is the first identification of a crucial role for TMEM106A in HCC.

Epigenetic plasticity is a pivotal driver of tumor metastasis. Aberrant promoter hypermethylation at CpG sites associated with tumor suppressor genes (ZNF471 [32] and FBXL7 [33]) can silence transcription and promote carcinogenesis. In HCC, several studies have demonstrated that tumor suppressor genes are frequently inactivated by methylation [ 34, 35] . Herein, we observed a decreased expression of TMEM106A in highly metastatic hepatoma cells, which was related to TMEM106A promoter methylation and was reversed by treatment with a demethylating agent, 5-Aza-2′-deoxycytidine. In addition, we found that 83% of HCC patients had a methylated TMEM106A CpG island. Hypermethylation was found to be related to the decreased TMEM106A mRNA and protein levels. MEXPRESS revealed a negative relationship between the degree of TMEM106A promoter methylation and TMEM106A expression. Clinically, TMEM106A methylation can discriminate tumor tissues from non-malignant adjacent tissues of HCC patients. Similar to these results, TMEM106A methylation was detected in 88.6% of primary gastric cancer tissue samples and in 18.1% of normal gastric tissue samples [36], suggesting its potential diagnostic clinical value.

DNA methylation alterations have been associated with clinico-pathological tumor aggressiveness and poor clinical outcomes ( *e*. *g*., DAPK and ZNF132) [ 37, 38] . In this study, we found that TMEM106A hypermethylation is associated with tumor size. However, TMEM106A hypermethylation is not related to vascular invasion, differentiation, or TNM stage of the tumor. Significantly negative relationships were observed among TMEM106A methylation, OS, and PFS in HCC patients, which encourage us to verify these results in a larger clinical population. To our knowledge, this is the first indication that evaluation of TMEM106A hypermethylation may be of diagnostic and/or prognostic value in cancer patients.

To assess whether molecular mechanisms other than methylation underlie the transcriptional regulation of TMEM106A, we investigated single nucleotide variants (SNVs) as well as homozygous and heterozygous deletions using the TCGA database of HCC patients. In HCC patients, only 0.2% had TMEM106A SNVs. No homozygous and heterozygous TMEM106A deletions were detected ( Supplementary Figure S2). These data suggest that hypermethylation of the TMEM106A promoter is the principal mechanism by which TMEM106A is silenced.

To our knowledge, the molecular mechanisms underlying the antitumor effect of TMEM106A remain poorly described. An important result of this study is that TMEM106A knockdown promotes HCC progression by inducing EMT via Erk1/2/Slug signaling. The importance of EMT is its close association with various processes of tumor development, including tumor occurrence, tumor stemness, distant metastasis, and drug resistance. EMT is often regulated by several cellular signaling pathways. This study demonstrated that HCC cells with lower TMEM106A expression showed higher expressions of Vimentin and N-cadherin and lower expressions of E-cadherin, indicating that TMEM106A may be an effective EMT inhibitor which contributes to less invasiveness and metastasis of HCC cells. The loss of the major epithelial marker, E-cadherin, an adhesion junction protein encoded by CDH1, is often considered as a hallmark of EMT. Three different types of transcriptional repressors, including the Snail, ZEB, and Twist families, are widely known as EMT-transcription factors (EMT-TFs) which play key roles in EMT-induced cancer progression through direct binding to the promoter region of *CDH1*. In this study, Slug, but not ZEB or Twist, was found to be critical to TMEM106A-mediated EMT and HCC malignant phenotype. Several studies have demonstrated that EMT-TFs are regulated by activation of cellular signaling pathways, including the PI3K/AKT/mTOR pathway, Wnt signaling pathway, RAS/RAF/MEK/Erk (MAPK) pathway, and the transforming growth factor-β (TGF-β) signaling pathway [ 25– 27] . These pathways can regulate the epithelial and mesenchymal character of cancer cells by regulation of EMT-TF expression. Herein, mechanistic analysis demonstrated that activation of the Erk1/2 /Slug signaling pathway is responsible for the progressive behavior of HCC cells regulated by TMEM106A knockdown ( Figure 8D). Similar to our results, other studies reported that inactivation of TMEM106A in macrophages enhances M1 polarization and induces inflammation via the MAPK signaling pathway [ 39, 40] , suggesting an important role for TMEM106A in the activation of the MAPK pathway.

Nevertheless, the present study has several limitations that require discussion. First, the molecular mechanism by which TMEM106A inhibits Erk1/2 phosphorylation was not completely elucidated. Future investigations will address this question. Second, whether demethylation treatment or DNA methyltransferase inhibitors exerts relevant anticancer effects through induction of TMEM106A expression and inhibition of EMT were not assessed. Further studies are needed to clarify this issue.

In summary, we demonstrate that tumor-specific methylation of TMEM106A frequently occurs in HCC and that such methylation plays an important role in the transcriptional regulation of TMEM106A. Furthermore, *in vitro* and *in vivo* analyses demonstrate that TMEM106A knockdown promotes HCC progression by inducting EMT through the Erk1/2/Slug signaling pathway which may serve as a potential therapeutic target.

## Supplementary Data

Supplementary data is available at *Acta Biochimica et Biophysica Sinica* online.
