# Corrigendum to: TMEM106A transcriptionally regulated by promoter methylation is involved in invasion and metastasis of hepatocellular carcinoma

**DOI:** 10.3724/abbs.2022069C

**Published:** 2024-06-05

**Authors:** Shiming Shi, Biao Wang, Jinglei Wan, Lina Song, Guiqi Zhu, Junxian Du, Luxi Ye, Qianqian Zhao, Jialiang Cai, Qing Chen, Kun Xiao, Jian He, Lei Yu, Zhi Dai

*Acta Biochim Biophys Sin* 2022, 54(7): 1008–1020 

https://doi.org/10.3724/abbs.2022069

In the version of this article initially published, an error was found in Figure 5D. The correct figure is as follows, and the interpretation of the results should be amended as follows: Matrigel invasion assay demonstrated that HepG2-shTMEM106A and PLC/PRF/5-shTMEM106A cells had increased invasive capacity compared with the corresponding control cells (46.0±5.5 vs 20.5±6.0, *P*=0.001; 29.0±2.6 vs 10.0±1.8, *P*<0.001, respectively; Figure 5D). The authors apologize for this error and any confusion it may have caused.

**Figure 1:**
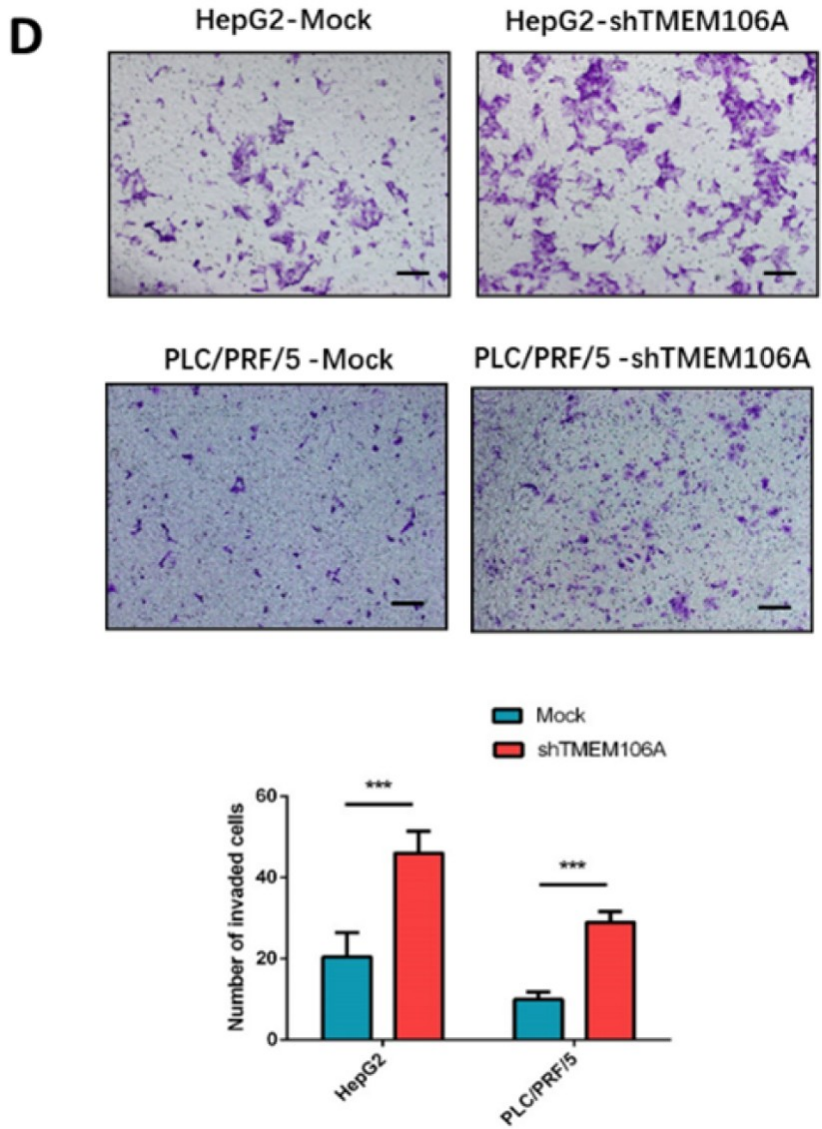
Figure 1

